# Toxin-Independent Virulence of *Bacillus anthracis* in Rabbits

**DOI:** 10.1371/journal.pone.0084947

**Published:** 2014-01-08

**Authors:** Haim Levy, Itai Glinert, Shay Weiss, Assa Sittner, Josef Schlomovitz, Zeev Altboum, David Kobiler

**Affiliations:** Department of Infectious Diseases, Israel Institute for Biological Research, Ness Ziona, Israel; Duke University Medical Center, United States of America

## Abstract

The accepted paradigm states that anthrax is both an invasive and toxinogenic disease and that the toxins play a major role in pathogenicity. In the guinea pig (GP) model we have previously shown that deletion of all three toxin components results in a relatively moderate attenuation in virulence, indicating that *B. anthracis* possesses an additional toxin-independent virulence mechanism. To characterize this toxin-independent mechanism in anthrax disease, we developed a new rabbit model by intravenous injection (IV) of *B. anthracis* encapsulated vegetative cells, artificially creating bacteremia. Using this model we were able to demonstrate that also in rabbits, *B. anthracis* mutants lacking the toxins are capable of killing the host within 24 hours. This virulent trait depends on the activity of AtxA in the presence of pXO2, as, in the absence of the toxin genes, deletion of either component abolishes virulence. Furthermore, this IV virulence depends mainly on AtxA rather than the whole pXO1. A similar pattern was shown in the GP model using subcutaneous (SC) administration of spores of the mutant strains, demonstrating the generality of the phenomenon. The virulent strains showed higher bacteremia levels and more efficient tissue dissemination; however our interpretation is that tissue dissemination *per se* is not the main determinant of virulence whose exact nature requires further elucidation.

## Introduction

The tripartite toxin and the poly-D-glutamic acid capsule are considered the major virulence factors of *Bacillus anthracis*, the etiological agent of anthrax. The capsule, composed of poly-D-glutamic acid and encoded by the pXO2 plasmid, allows unrestrained bacilli growth in the infected host, since it inhibits phagocytosis of the vegetative cells by the innate immunity system (macrophages and neutrophils). The tripartite toxin consists of lethal factor (LF), edema factor (EF) and protective antigen (PA), (encoded by *lef, cya* and *pag* genes respectively, located in a pathogenicity island on the pXO1 plasmid). Toxic activity is expressed only when PA is combined with LF (forming the lethal toxin, LT) or EF (forming the edema toxin, ET) [1]. *In vitro*, toxin and capsule production are optimal when cells are grown in inductive conditions (CO_2_ and 37°C) reflecting the normal mammalian host environment [1]. AtxA, a *B. anthracis* global regulator, encoded by *atxA* and located in the pathogenicity island on pXO1, was shown to mediate the interaction between environmental conditions and the metabolic state of the bacterium and the expression of the virulence factors [3], [4], [5].

Curing pXO1 from *B. anthracis* results in complete loss of virulence, indicating the possible essential role of the toxins in pathogenicity. In previous studies we evaluated the assumption that LT and ET play major roles in the pathogenicity of *B. anthracis* by a systematic genetic study deleting the *pag*, *lef* and *cya* genes and their combinations [6], [7], [8]. The effects of the mutations on virulence were tested in rabbits and guinea pigs (GP) using two routes of infection, subcutaneous injection (SC) and intranasal instillation (IN). The results demonstrated that while the toxins are necessary for optimal virulence (full mortality and wild type mean time to death) in all models tested, one toxin is sufficient for efficient pathogenicity. These results were corroborated in a rabbit model of inhalational anthrax [9].

In the rabbit model, the toxins play a major role in virulence, as the deletion of either *pag* alone or *lef* and *cya* completely attenuates the strain when introduced IN or SC, similar to the effect of pXO1 curing. On the other hand, in the GP model no major role could be attributed to the toxins. In GP, deletion of *pag* or the three toxin components resulted only in moderate attenuation and prolonged mean time to death (MTTD), whereas the pXO1-cured mutant showed complete attenuation. These results may indicate that *B*. *anthracis* possesses an additional virulence mechanism, exhibited in GP, which is toxin independent but pXO1 dependent [6].

In previous studies we and others have suggested that toxins play a major role in the early stages of the infection, enabling the organism to overcome innate host defenses, whereas the death of the animals relates to bacteremia and organ bacterial burden rather than systemic toxemia [6], [9], [10]. This assumption was further supported by the finding that passive immunization with anti-PA antibodies could prevent the establishment of disease in animals exposed to *B. anthracis* spores, but could not cure (and save) bacteremic animals [11], [12], [13], [14].

To characterize the toxin-independent virulence trait as a possible cause of death, we established a new rabbit model; artificially creating bacteremia by intravenously injecting *B. anthracis* encapsulated vegetative cells. In this manuscript we show using this model that in rabbits, similarly to previous data from GP, *B. anthracis* mutants lacking the toxins, even though partially attenuated still maintain significant virulence, killing the host within 24 hr. We also demonstrate that this toxin independent virulence trait depends on the activity of AtxA in the presence of pXO2, as deletion of either component abolishes virulence. The same pattern was shown in the GP model using SC administration of spores of the mutant strains, demonstrating the potential generality of the phenomenon.

## Materials and Methods

### Bacterial strains, media and growth conditions

Bacterial strains used in this study are listed in **Table 1**. *B. anthracis* and *Escherichia coli* strains were cultivated in Terrific broth [15] at 37°C with vigorous shaking (250 rpm). For the induction of toxins and capsule production, a modified DMEM (supplemented with 10% normal rabbit serum, 4 mM L-glutamine, 1 mM sodium pyruvate, 1% non-essential amino acid) was used. Sporulation was carried out using G broth, as previously described [16]. *E. coli* strains were used for the facilitation of plasmid construction. Antibiotic concentrations used for selection in Mueller Hinton (MH) agar (Difco)/Terrific broth were: for *E. coli* strains, ampicillin (Amp, 100 µg ml^−1^); for *B. anthracis* strains, kanamycin (Kn, 10 µg ml^−1^), and erythromycin (Ery, 5 µg ml^−1^).

**Table 1:** Bacterial strains, plasmids and oligonucleotide primers used in this study.

	Description/characteristics	Source
Strain		
***B. anthracis***		
Vollum	ATCC 14578	IIBR collection
VollumΔ*pag*Δ*cya*Δ*lef*	Complete deletion of the *pag*, *lef* and *cya* genes	[6]
VollumΔ*pag*Δ*cya*Δ*lef*Δ*atxA*	Complete deletion of the *atxA* gene in the VollumΔ*pag*Δ*cya*Δ*lef* mutant	This study
VollumΔ*pag*Δ*cya*Δ*lef*Δ*bslA*	Complete deletion of the *bslA* gene in the VollumΔ*pag*Δ*cya*Δ*lef* mutant	This study
VollumΔpXO1*BA2805*::*atxA*	Genome insertion of the *atxA* gene replacing major parts of the PlyPH (*BA2805*)[31] in the VollumΔpXO1	This study
VollumΔ*pag*Δ*cya*Δ*lef*ΔpXO2	Curing of the pXO2 plasmid in the VollumΔ*pag*Δ*cya*Δ*lef* mutant	This study
VollumΔpXO1	Vollum pXO1−, pXO2+	IIBR Collection
VollumΔpXO2	Vollum pXO1+, pXO2−	IIBR Collection
VollumΔpXO1ΔpXO2	Vollum pXO1−, pXO2−	IIBR Collection
***E. coli***		
DH5α	*endA1 recA1*	IIBR collection
GM2929	dam::Tn9 (CmR) dcm-6	NEB
		
Plasmids		
pEGS	Allelic replacement vector platform	[8]
pEGS-atxA		This study
pEGS-bslA		This study
pEGS-BA2805::atxA		This study
pEGS-CAP		This study
Primer	Sequence a	Complementary Position (**A**UG = 1)
ATX1	TCTTCAATGTCTTGTAAATTAATT	−550
ATX2	tttgcggccgcTTTCTCCTGGCTTTCTTTTAGGTA	−410
ATX3c	tgtactagtGTCTATAATTGATTCTCCTTTCCT	−23
ATX4	ataactagtATGCCCTTTAAATATTTGTTTAAT	1428
ATX5c	ggcgcgccATAAAAACGACATATAAATATGTC	1900
ATX6c	CTCAATAAACTCAAAACTAATTGT	2119
ATXs1	ATTAATTTACTACACTTTATCAAT	42
ATXs2	CAGTTTCATGTAATGTAACGCCGA	742
PX901	GTGGGTTAAATGGTGG	−482
PX902	gacgcgcggccgcAGGATATGCCCACG	−430
PX903c	ggagtagtGCGTTTTCTCTGTGTGC	−39
PX904	ggactagtGTAACCCTAAACC	1280
PX905c	ttggcgcgccCATATATAATAGTACCTCC	2230
PX906c	AACGTTTCACTTGCC	2332
2805 2	gacgcgcggccgcAAAGCACGGCTACCG	−287
2805 3c	ggactagtCCCATAACTTAACACCTCC	5
2805 4	ggactagtTTTCGGTATGG	381
2805 5c	ttggcgcgccCGCTCCCATAACATCTGGTG	663
ATXcomp1	ataactagtTATACTCACCAAAAATTTCAAGGT	−913
ATXcomp2c	ataactagtTTATATTATCTTTTTGATTTCATG	27 past ter.

^a^ The homology region to the coding sequence is marked in capital letters.

### Plasmid and strain construction

Plasmids and oligonucleotide primers used in this study are summarized in **Table 1**. The oligonucleotide primers were designed according to the genomic sequence of *B. anthracis* Sterne strain. Genomic DNA (containing the chromosomal DNA and the native plasmids, pX01 and pX02) for cloning the target gene fragments was extracted from the Vollum strain as previously described [6]. Polymerase chain reaction (PCR) amplifications were performed using the AccuTaq LA systems (Sigma).

Prior to transformation into the Vollum strain, all plasmids were first propagated in the methylation deficient *E. coli* strain GM2929 (**Table 1**). *B. anthracis* cells were electrotransformed as described [18].

Target genes were disrupted by homologous recombination, using a previously described method [8], [19]. In general, gene deletion was accomplished by a marker-less allelic exchange technique that replaced the complete coding region with the *Spe*I restriction site. At the end of the procedure the resulting mutants did not code for any foreign sequences and the only modification is the null mutation of the target gene. To construct a VollumΔpXO1 strain that encodes a genomic copy of the *atxA* gene, we used the plasmid pEGS 2805, which we previously used to inactivate the BA2805 in the wild type Vollum strain (no effect in virulence in guinea pigs, data not shown). The *atxA* gene with the upstream ORF were amplified using primers ATXcomp1 and ATXcomp2c and cloned into the *Spe*I site of the pEGS2805 located between the sequences with homology to the 5′ (primers 2805 2-3c) and the sequences homologous to the 3′ (primers 2805 4-5c) of the BA2805 gene. The different mutations were verified by PCR for the deletion of the target gene, and that no major rearrangements occurred in the area of the deletion. All the mutants were tested for their ability to produce capsule by incubation in modified DMEM in 10% CO_2_ atmosphere. The capsule was visualized by negative staining with India ink.

To cure the pXO2 from the VollumΔ*pag*Δ*lef*Δ*cya* mutant, the pEGS CAP was inserted, by single cross over into the *capA* – *capD* region of the pXO2 plasmid (primers 5′ gacgcgcggccgcCAAGGGGGTGAGAGG and 3′ ttggcgcgccGGGGCAGATATTATTGTGG). The resulting GFP positive clone was cultured in 2 ml terrific broth in 15 ml falcon tube at 40°C 250 rpm. Every 24 hr 1 µl of the culture was plated on LB plate and the culture was diluted 1:20 into fresh media. Dark non florescent colonies were scored following over night incubation at 37°C. These colonies were tested for erythromycin sensitivity and the presence of pXO1 and absence of pXO2 by PCR. The absence of pXO2 was confirmed by the absence of a typical capsule following induction, as previously described. The genotype and phenotype of the VollumΔpXO1 or ΔpXO2 were verified by PCR, capsule production and ELISA for the secretion of PA.

In *in-vitro* studies we demonstrated that the growth and sporulation profiles (data not shown) as well as capsule production of all mutants were indistinguishable from those observed for the parental wild type Vollum strain

### DNA preparation and PCR

DNA was purified from *Bacillus* cultures or colony collections as described previously [17]. For fast colony screening, each colony was resuspended in 50 µl of sterile double distilled water (DDW) in a 0.2 ml PCR tube. The tube was then placed in a thermocycler for two cycles of 95°C for 10 min. The tubes were then centrifuged in a minifuge, at maximal speed for 1 min at room temperature. The clear supernatant was transferred to a clean tube and 5 µl were used for the PCR reactions.

All PCR reactions (25 µl) were performed in 1xPCR buffer (3.5 mM MgCl2); dNTPs (0.2 mM each); 0.04 U/μl of TaKaRaTaq DNA polymerase (all from TaKaRa Bio Inc. R001A) and ∼2 ng of DNA. The general thermocycling program for the PCR reaction was 95°C for 30 sec followed by 40 cycles of 94°C for 1 min; 55°C for 30 sec; 72°C for 1 min, and then one cycle of 72°C for 5 min. The PCR products were separated on 1.1% agarose gel using 1× TBE as running buffer.

### Infection of rabbits and guinea pigs

Female New Zealand white rabbits (Charles River Laboratories or Harlan Laboratories), 2.2–2.5 kg were used to test the virulence of the wild-type and mutant Vollum strains. Spores were germinated by incubation in Terrific broth for 1 hr at 37°C, and then incubated in modified DMEM in 10% CO_2_ atmosphere for 2 hr at 37°C, to induce capsule formation. The capsule was visualized by negative staining with India ink. The capsulated vegetative cells were injected IV via the ear vein and a remaining sample was plated for total viable counts (CFU/ml). The animals were observed daily for 14 days or for the indicated period. Upon death, blood samples were plated and DNA was extracted, followed by PCR analysis in order to determine the identity of the strain responsible for the animals' death.

Female Hartley guinea pigs (Charles River Laboratories), weighing 220–250 g were used. The animals were infected with spore preparations of either the mutant strains or the parental Vollum strain. Prior to infecting the animals, the spore preparations were heat-shocked (70°C, 20 min) and serially diluted in saline to produce spore suspensions within the range 10^2^–10^9^ per milliliter. A spore dose of 0.1 ml was administered subcutaneously (SC) to each animal. The remaining spore dose suspensions were plated for total viable counts (CFU/ml). The animals were observed daily for 14 days or for the indicated period. Upon death, blood samples were plated and DNA was extracted, followed by PCR analysis in order to confirm the identity of the strain responsible for the animals' death.

This study was carried out in strict accordance with the recommendations of the Guide for the Care and Use of Laboratory Animals of the National Research Council. The protocols were approved by the Committee on the Ethics of Animal Experiments of the IIBR (permit numbers GP-08-2012, RB-06-2012, RB-24-2012, RB-25-2013). Animals were euthanized when one of the following symptoms was detected: severe respiratory distress or the loss of righting reflex. Guinea pigs were sacrificed by CO_2_ inhalation and rabbits by the sodium pentabarbitone injection.

### Determination of bacterial burden in tissues

Infected rabbits were euthanized either 5 or 24 hr post-infection with Pental (sodium pentobarbitone) and various organs were harvested. The organs: spleen, lungs, brain, kidneys and liver, were immediately homogenized and serial dilutions of the homogenate were plated on agar plates to determine the bacterial level.

### Production of convalescent sera and passive immunization

Convalescent sera were prepared as previously described [21]. Rabbits were intranasally inoculated with 10^7^ Vollum spores. Thirty hours later the rabbits were bled for bacteremia determination and immediately afterwards antibiotic treatment (ciprofloxacin) was initiated. Rabbits that were bacteremic at the beginning of treatment and survived the antibiotic treatment were bled 30 days post inoculation and the convalescent serum was frozen till use. Rabbits prior to inoculation and rabbits that did not show any bacteremia at the beginning of treatment were bled to prepare control sera.

For passive immunization, 20 ml of sera were injected IV to naïve rabbits 2 hours prior to IV inoculation with capsulated vegetative Vollum wild type or mutant cells. Since the challenge strain is missing the toxins – LF, EF and PA, the presence of antibodies against these proteins does not have any neutralizing effect.

### Protein extraction and Immunoblotting

Spores (2.5×10^7^ CFU) were germinated by incubation in Terrific broth for 1 hr at 37°C, and then incubated in 10 ml modified DMEM in 10% CO_2_ atmosphere for 16 hr at 37°C. The culture pellet was resuspended in 0.5 ml of 6× protein loading buffer and incubated of 15 min in 99°C. 20 µl of the clear supernatant was loaded on 10% SDS PAGE, transferred to PVDF membrane and blotted with specific antisera.

### Statistical analysis

The vegetative bacteria lethal dose required to kill 50% of the animals (LD_50_) was calculated by the method of Reed and Muench [20]. The bacteremia and bacterial organ load were grouped according to the sample time (5 or 24 hr post infection) and whether the infection was lethal or not. The groups were plotted as the distribution of each of the animals in the group (scattered). The significance of the differences in the blood bacterial burden between the virulent and non virulent mutants was determined by t test comparing the log10 values of the CFU/ml, using GraphPad Prism version 5.00 for Windows (GraphPad Software, San Diego California USA, www.graphpad.com). The mean time to death (MTTD) was calculated for each mutant as the sum of the days till death of all the animals that succumbed divided by their number. No score was given to animals that survived the infection.

## Results

### Establishment of the IV inoculation model

**Figure 1:**
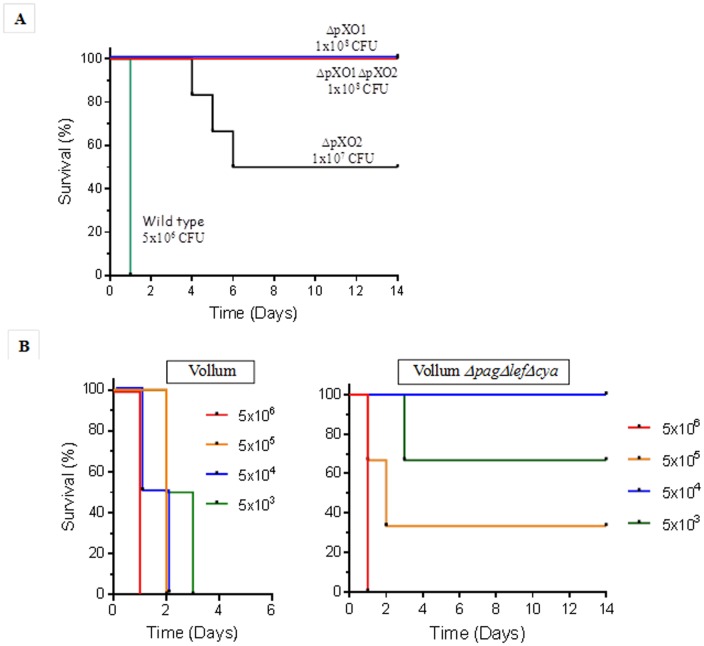
Virulence of mutants or wild type Vollum strains following IV inoculation of rabbits. Rabbits were inoculated IV with vegetative cells of the wild type and mutants strains. **A**. Survival of rabbits inoculated with the fully virulent Vollum (n = 4), the capsular VollumΔpXO1 (n = 4), toxinogenic VollumΔpXO2 (n = 6) or the non-capsular non-toxinogenic VollumΔpXO1ΔpXO2 strains (n = 4). Strain name and inculcation doses are as marked. **B**. Survival of rabbits inoculated with different doses of the wild type Vollum (n = 2 to 4) or the VollumΔ*pag*Δ*lef*Δ*cya* (n = 3 to 4) strains (also see **Table S1**).

To characterize the toxin-independent virulence trait as a possible cause of death, we established an IV inoculation model in rabbits. Vegetative encapsulated bacilli were cultured in inductive medium (M&M) and injected IV to test their ability to cause lethal disease. Upon IV inoculation, the encapsulated vegetative bacteria disperse and are diluted in the circulation. The bacterial dose was calculated, according to an estimate of blood volume of 10% of the body weight, to a final concentration in the range of 10^4^ CFU/ml, that we have previously shown to be treatable by antibiotics [21]. In reality, 30 minutes post inoculation the bacteremia detected was about a tenth of the expected concentration (data not shown), suggesting rapid clearance from circulation. Additional doses were used for the different mutants depending on the strains' virulence. The results, shown in **Figure 1A**, demonstrate that injection of the fully virulent Vollum strain (pXO1+pXO2+) caused death within 24–48 hr, whereas the VollumΔpXO1ΔpXO2 strain was completely avirulent in the rabbit model. IV inoculation of encapsulated vegetative Vollum bacilli resulting in bacterial levels of less than 1 CFU/ml (total dose of ∼10 CFU) is sufficient to cause lethality in rabbits (**Table S1**).

### Are *B. anthracis* toxins essential for virulence?

Using the IV inoculation model, we evaluated the involvement of pXO1 and the toxins in the bacterial capacity to cause death in inoculated rabbits. As can be seen in **Figure 1A**, curing pXO1 from *B. anthracis* results in the complete loss of virulence. On the other hand, the mutant strain lacking toxins but containing pXO1 (VollumΔ*pag*Δ*cya*Δ*lef*) shows significant virulence (**Figure 1B**), efficiently killing the IV inoculated rabbits. This mutant exhibited strong attenuation compared to the wild type (a difference in LD50 of >4 orders of magnitude; LD_50_mut = 2×10^5^ vs. LD_50_wt = <10, **Table S1**), requiring a higher dose of encapsulated bacteria to cause mortality, while presenting a longer MTTD. This result is corroborated by a previous report stating that a Δ*pag* mutant shows full attenuation using IV inoculation of 10^5^ bacilli [9].

**Figure 2:**
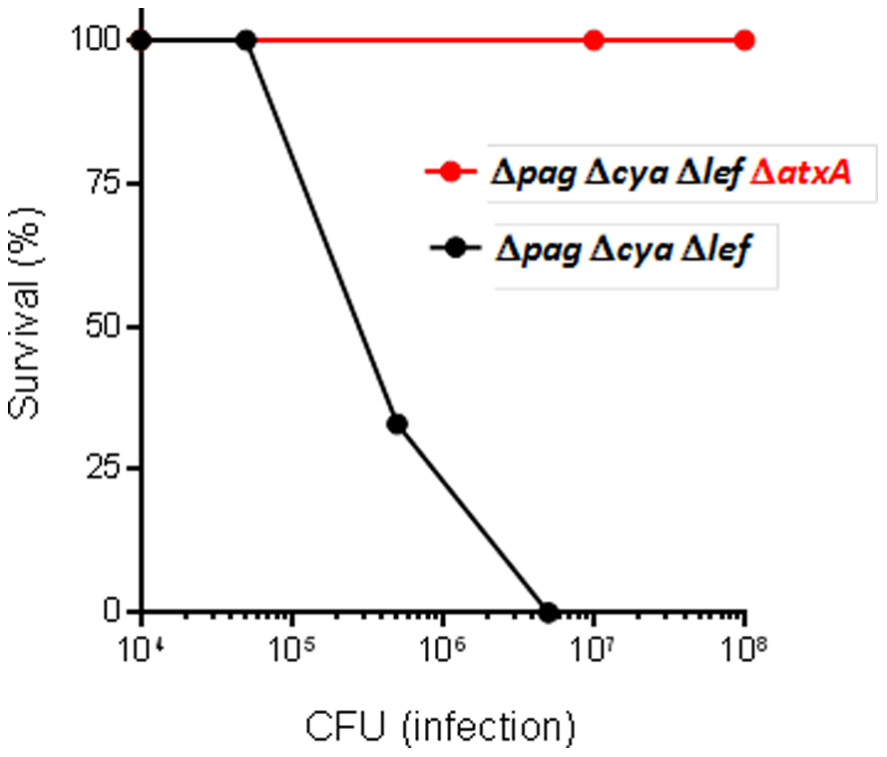
The toxins-independent virulence is AtxA dependent. Rabbits were inoculated IV with different doses of vegetative cells from the VollumΔ*pag*Δ*lef*Δ*cya* (n = 3 to 4) or VollumΔ*pag*Δ*lef*Δ*cya*Δ*atxA* (n = 4) mutant strains (also see **Table S1**).

The finding that virulence is dependent upon the presence of pXO1, but does not require the toxins, led us to test the involvement of AtxA, the *B. anthracis* global activator, in this phenomenon. Deletion of the *atxA* gene, as in the VollumΔ*pag*Δ*cya*Δ*lef*Δ*atxA* strain, completely abolishes lethality (**Figure 2**). This finding demonstrates the major role AtxA has in the regulation of the virulence factors that function in this modality. Deletion of pXO2 from the VollumΔ*pag*Δ*cya*Δ*lef*, results in the creation of a non-virulent strain (VollumΔpXO2Δ*pag*Δ*cya*Δ*lef*) as demonstrated by IV inoculation of rabbits (**Table S1**) and SC spore inoculation of guinea pigs (**Table 2**). This may indicate that AtxA-dependent virulence results from regulation of pXO2-based elements. On the other hand, these results can be interpreted as if pXO2 supports bacterial survival in the circulation, allowing AtxA to function and to exhibit the virulent trait.

**Table 2:** Susceptibility of GP to *B. anthracis* strains in the SC injection model.

Strain	Description	Inoculum (CFU)	Dead/infected	MTTD (days)
Vollum	pXO1^+^pXO2^+^	10^3^	6/6	3
				
Vollum ΔpXO1ΔpXO2	pXO1^−^pXO2^−^	10^8^	0/8	>14
				
Vollum ΔpXO1	pXO1^−^pXO2^+^	10^8^	0/5	>14
				
Vollum Δ*pag*Δ*cya*Δ*lef*	Complete deletion of the *pag, lef* and *cya* genes	10^6^	2/3	2.5
		10^5^	4/6	5.75
				
Vollum Δ*pag*Δ*cya*Δ*lef*Δ*atxA*	Complete deletion of the *pag, lef*, *cya* and *atxA* genes	10^8^	0/4	>14
		10^7^	0/4	
				
Vollum ΔpXO2Δ*pag*Δ*cya*Δ*lef*	Complete deletion of pXO2 and the *pag, lef* and *cya* genes	10^8^	0/4	>14
				
Vollum ΔpXO2	pXO1^+^pXO2^−^	10^8^	4/5	7.75
		10^7^	0/5	>14

For the sake of completeness, the toxinogenic non-capsulated strain, VollumΔpXO2, or VollumΔ*atxA* which are completely attenuated when administrated SC, were also tested using this model. As can be seen in **Figure 1A** and **Table S2**, both mutants caused the death of 50% of the injected rabbits, with a longer MTTD.

**Figure 3:**
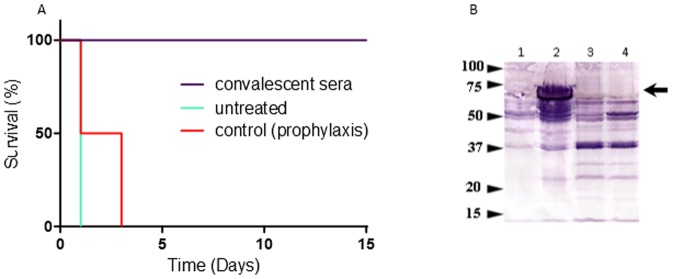
Passive protection of rabbits using convalescent sera against IV challenge with the VollumΔ*pag*Δ*cya*Δ*lef* mutant. **A**. 20 ml of sterile convalescent or control sera were administered IV to rabbits (n = 4) three hours before an IV challenge with 5×10^6^ CFU of the vegetative VollumΔ*pag*Δ*cya*Δ*lef* mutant cells. **B**. Cellular antigens recognized by the protective sera. Proteins were extracted by boiling of different encapsulated *B. anthracis* mutants. The blots were immune-blotted by a representative protective serum. 1. VollumΔpXO1. 2. VollumΔ*pag*Δ*cya*Δ*lef*. 3. VollumΔ*pag*Δ*cya*Δ*lef*Δ*atxA*. 4. VollumΔ*pag*Δ*cya*Δ*lef*Δ*bslA*. The size marker in kDa is marked on the left. The arrowhead marks the location of the BslA protein.

In an additional attempt to elucidate the underlying virulence mechanism, exploring the possibility of protection using antibodies, we attempted passive immunization using sera from convalescent rabbits (material and methods). It is our experience that this serum contain antibodies against cellular and secreted components, conferring full protection to the convalescent rabbit from a challenge with the wild type Vollum strain spore [21]. As the deletion of all toxin components does not affect virulence, we assumed that the accepted PA-based vaccination (or passive immunization with sera from a PA-vaccinated animal) is not applicable in this model. While infecting naïve rabbits IV with 10^7^ VollumΔ*pag*Δ*cya*Δ*lef* encapsulated vegetative cells results in death within 24 hr, the described passive immunization with sera from convalescent rabbits (**Figure 3**A) was able to fully protect rabbits from the same infection dose. This passive protection was specific to convalescent sera since pretreatment with pre- immune sera (or sera from rabbits that were exposed to spores and prophylacticly treated with antibiotics) did not confer protection under the same IV vegetative cell challenge conditions (n = 4).

In a previous study [6] we have shown that GP are susceptible to the spores of the VollumΔ*pag*Δ*cya*Δ*lef* strain, similar to the finding with the rabbit IV model. Therefore, using the GP SC spore injection model, we decided to test whether a similar dependence of virulence on AtxA activity can be demonstrated. As can be seen in **Table 2**, there is a clear parallel pattern in the virulence exhibited by the various Vollum strains in the IV rabbit model and the GP SC model. In both models, the virulence of the Vollum strains is dependent on the presence of *atxA* on the pXO1 plasmid, but does not require the toxins. Here again, it seems that both AtxA and the pXO2 plasmid are required for the efficient exertion of virulence, as deletion of pXO2 abolishes virulence (**Table 2**, **Table S1**).

These results raised the question whether the difference between rabbit and GP reflects a fundamental difference between administration routes or rather a sensitivity difference between animal models. In order to clarify this point we tested the susceptibility of rabbits to SC injection of high doses of VollumΔ*pag*Δ*cya*Δ*lef* spores. Whereas a dose of 10^7^ CFU spores injected SC did not kill the infected rabbits (0/8), an inoculum of 10^8^ VollumΔ*pag*Δ*cya*Δ*lef* spores resulted in a lethality rate of 75% (6/8). Therefore, we can conclude that while GP are more susceptible than rabbits, the basic mechanisms can be demonstrated in both models.

### Does capsule production and function reflect pXO2 activation?

**Figure 4:**
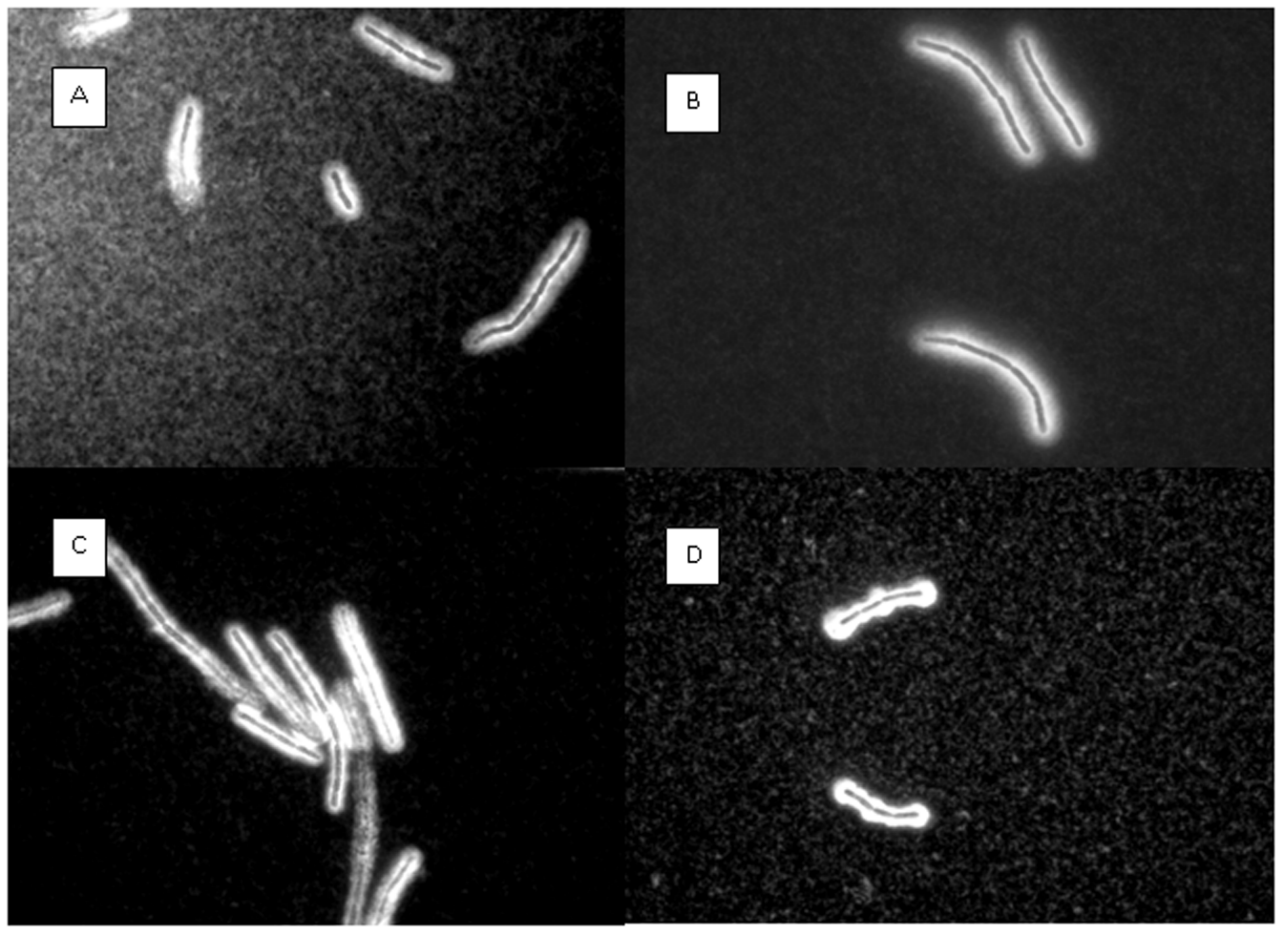
Capsule production by the different mutants. Vegetative bacteria were incubated for 2 hs in inductive medium, and the capsules were negatively stained with India ink. No difference could be detected between virulent strains and non-virulent strains. (A) Vollum-wt, (B) VollumΔ*pag*Δ*cya*Δ*lef*, (C) VollumΔ*pag*Δ*cya*Δ*lef*Δ*atxA,* (D) VollumΔpXO1.

The observed differences in virulence between the Vollum strains may be related to AtxA exerting its activity on pXO2. The main function of pXO2 in virulence is assumed to be the generation of the capsule, which inhibits phagocytosis of vegetative cells by innate immune system cells, like macrophages and neutrophils. Therefore we compared the various *B. anthracis* strains for their ability to produce/generate the capsule, and the survival of their encapsulated vegetative cells in the circulation. As can be seen in **Figure 4**, no qualitative difference could be detected in capsule morphology (following two hours incubation in the inductive growth media) between the virulent strains Vollum-wt and VollumΔ*pag*Δ*cya*Δ*lef*, and the non-virulent strains, VollumΔ*pag*Δ*cya*Δ*lef*Δ*atxA* and VollumΔpXO1.

**Figure 5:**
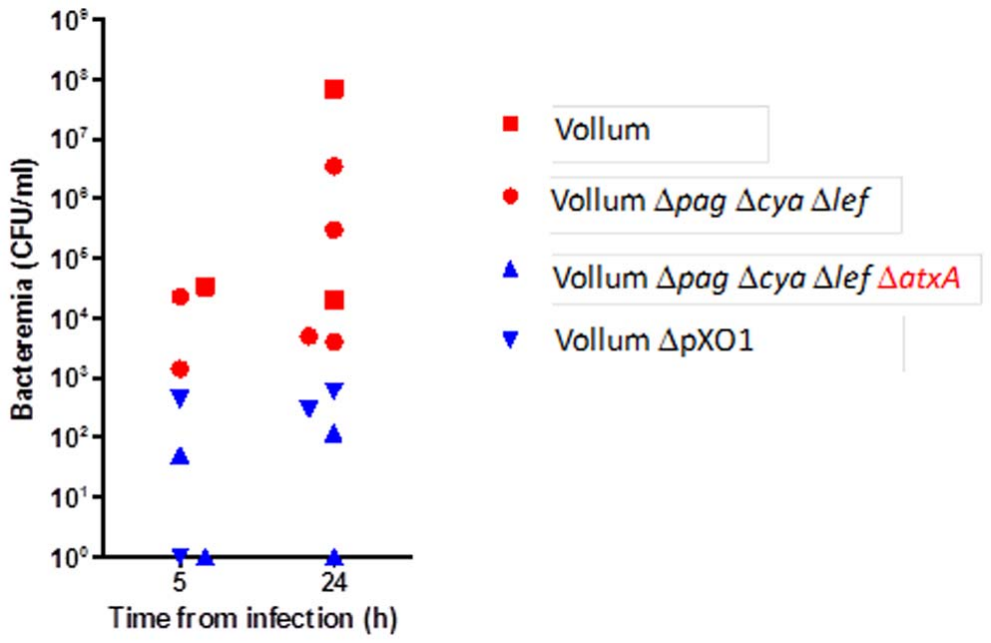
Bacteremia following intravenous injection of vegetative cells of the different mutants. Vegetative capsulated bacteria (10^7^) were inoculated IV and the bacteremia levels in individual animals, were determined 5 and 24 hr post injection. Virulent strains (Vollum-wt - red square and Vollum-Δ*pag*Δ*cya*Δ*lef* - red circle) showed higher bacteremia than non-virulent strains (VollumΔ*pag*Δ*cya*Δ*lef*Δ*atxA* - blue triangle and VollumΔpXO1 – inverted blue triangle).

On the other hand, determination of bacteremia in the rabbit circulation 5 hr and 22–24 hr post IV injection with inoculums of 10^7^ CFU (**Figure 5**) shows a clear tendency of the virulent strains (red shapes) to maintain higher bacterial levels (P = 0.0092) than the non-virulent strains (blue shapes). In all rabbits tested (**Figure 5**), both virulent strains -Vollum-wt and VollumΔ*pag*Δ*cya*Δ*lef*, showed higher bacteremia (>10^3^ CFU/ml) than the non-virulent strains - VollumΔ*pag*Δ*cya*Δ*lef*Δ*atxA* and VollumΔpXO1 (<10^3^ CFU/ml) at the two time points tested. Corroborating this finding, IV inoculation with VollumΔ*pag*Δ*cya*Δ*lef* encapsulated vegetative cells of rabbits passively immunized with sera from convalescent rabbits (using fully protective conditions, as described above), showed a decreased bacteremia, similar to the non-virulent strains. These differences in the levels of circulating bacteria could affect the dissemination of the bacteria to different host tissues.

### Does bacterial dissemination reflect variations in virulence?

**Figure 6:**
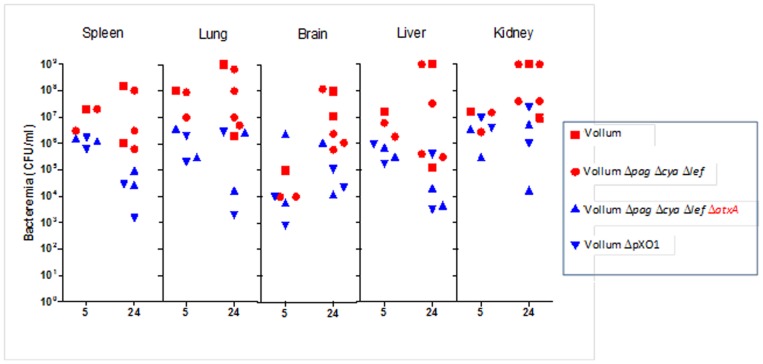
Organ bacterial load following intravenous injection of vegetative cells of the different mutants. Vegetative capsulated bacteria (10^7^) were inoculated IV and the bacterial burden, were determined 5 and 24 hr post injection at various tissues of individual animals. Virulent strains (Vollum-wt - red square and Vollum-Δ*pag*Δ*cya*Δ*lef* - red circle) showed a tendency to exhibit higher bacterial levels than non-virulent strains (VollumΔ*pag*Δ*cya*Δ*lef*Δ*atxA* - blue triangle and VollumΔpXO1 – inverted blue triangle).

To determine whether the observed differences in circulating bacteremia between the *B. anthracis* strains reflect a difference in their ability to spread systemically, a small-scale study was conducted to compare the bacterial burden in the different tissues at two different time points. Rabbits, IV inoculated with 10^7^ CFU of the Vollum strains, were sacrificed 5 hr and 24 hr p.i. and the bacterial content of the spleen, lungs, brain, liver and kidneys was determined. As can be seen in **Figure 6**, virulent strains seem to exhibit higher tissue bacterial burdens than the non-virulent strains, creating seemingly similar pattern to that seen in circulating bacteria levels.

These findings indicate that the virulence of *B. anthracis* strains may be related to their ability to spread to different tissues. Questioning the relevance of this assumption, we found that passive immunization with sera from convalescent rabbits, while saving naïve rabbits inoculated IV with VollumΔ*pag*Δ*cya*Δ*lef* encapsulated vegetative cells, did not seem to significantly affect bacterial tissue dissemination (data not shown).

Based on the findings of the passive immunization, an attempt was made to identify the antigen responsible for the protective activity of the sera of convalescent rabbits. Bacterial extracts were prepared from virulent and non-virulent strains and were compared by Western-blotting with protective sera. As can be seen in **Figure 3B**, the protective sera recognized among others, a distinct major band of about 70 Kd present in the VollumΔ*pag*Δ*cya*Δ*lef* extract but not in the Vollum ΔpXO1 and VollumΔ*pag*Δ*cya*Δ*lef*Δ*atxA* extracts. Deletion of the pXO1-90 (BslA – *B. anthracis* S-layer protein A), which is regulated by AtxA, from the VollumΔ*pag*Δ*cya*Δ*lef* resulted in the disappearance of this band from the Western-blot (**Figure 3B**). This mutation (Δ*bslA*) only slightly attenuated the pathogenicity of the mutant (**Table S1**) and did not affect tissue dissemination (data not shown). The finding that the toxin-independent virulent trait is not mediated by BslA is corroborated by the finding that AtxA is the only pXO1 gene required for the exhibition of this virulence (see below).

### Is pXO1 essential for the display of the toxin-independent virulence?

**Figure 7:**
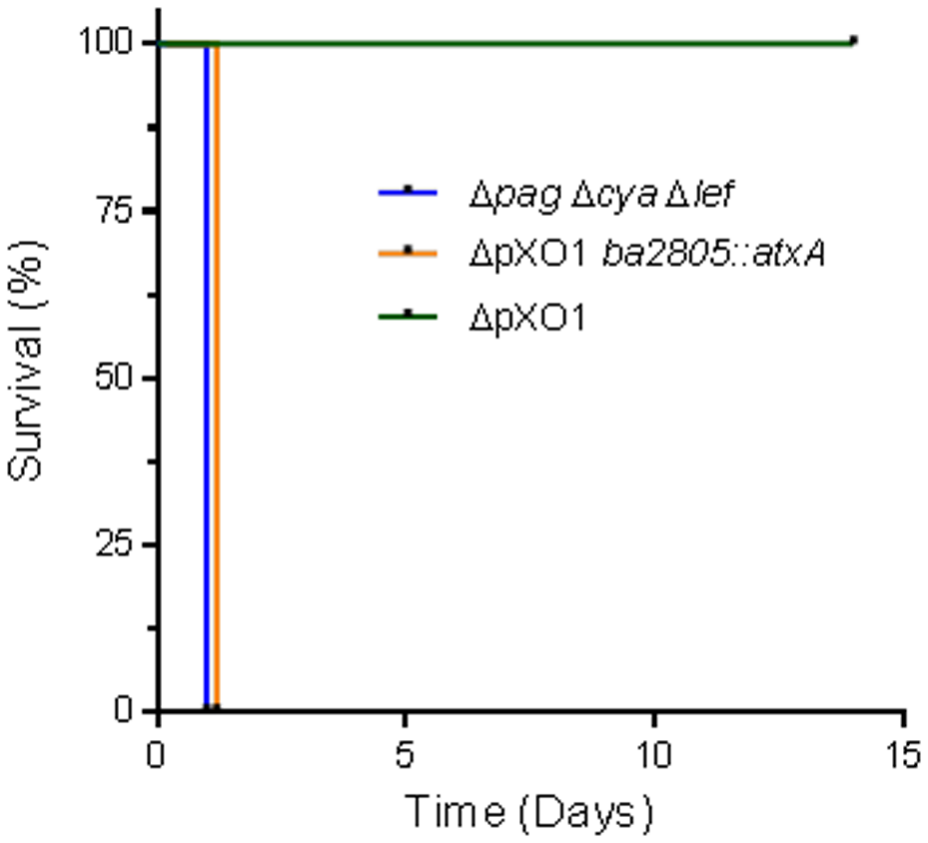
Genomic copy of *atxA* restores IV virulence to the VollumΔpXO1 mutant. The *atxA* gene with the necessary upstream regulatory elements were inserted into the VollumΔpXO1 strain replacing the BA2805 ORF. Rabbits (n = 4) were inoculated IV with 1×10^7^ CFU of vegetative cells of the different mutants (also see **Table S2**).

The toxin-independent virulent trait, exhibited following IV inoculation of encapsulated VollumΔ*pag*Δ*cya*Δ*lef* vegetative cells, depends on the activity of AtxA in the presence of pXO2, as deletion of either component abolishes virulence (**Figure 7**). This finding can be interpreted as if the AtxA-dependent virulence results from its regulation of pXO2-borne elements, rather than regulating pXO1- and genome-borne elements. The non-essential role of pXO1 in this virulent trait was demonstrated by inserting the *atxA* gene into the genome of the non-virulent Vollum ΔpXO1, creating the VollumΔ*pXO*1 *ba2805::atxA*. This insertion resulted in the recovery of the virulent trait (**Figure 7** **, Table S2**). These results demonstrate that *atxA*, in the background of pXO2, is sufficient for the exhibition of the toxin-independent virulent trait.

## Discussion

The accepted paradigm states that anthrax is both an invasive and toxinogenic disease and that the toxins play a major role in pathogenicity. This hypothesis was based mainly on studies carried out in mice using the attenuated Sterne strain (pXO1^+^pXO2^−^). In previous studies, we tested this assumption by a systematic genetic study deleting the toxin genes in a fully virulent strain, testing the virulence of the mutant strains in GP and rabbits. In both models, full virulence requires both toxins, LT and ET, but either one is sufficient for virulence [7], [8]. In the GP SC infection model, deletion of the all three toxin components, *pag, lef* and *cya* genes, results only in a relative moderate attenuation (approximately a hundred fold increase in LD_50_). These findings suggest that *B. anthracis* possesses an additional toxin independent virulence mechanism, since significant residual virulence is exhibited after fully deleting toxin components. Both mechanisms are pXO1 dependent, as the encapsulated non-toxinogenic (pXO1^−^pXO2^+^) mutant shows complete attenuation. On the other hand, rabbits were shown to be less sensitive to this toxin-independent virulence mechanism with toxins still required for the establishment of an effective (lethal) infection [6].

The ability of toxin-deficient mutants to effectively kill experimental hosts, coupled with abundant data regarding the function of these toxin on various systems, and especially the immune system, leads to the hypothesis that the toxins play a major role in the early stages of infection. These include the initial confrontation of the spores/bacteria with innate host defenses and their neutralization/evasion by the pathogen [10]. In order to test this hypothesis, we developed an assay designed to bypass these stages. Intravenous inoculation with encapsulated vegetative bacteria artificially creates bacteremia that resembles the septic stage of the disease, allowing the hematogenous spread of the bacilli and causing lethality in rabbits. IV injection of Vollum-wt encapsulated vegetative bacilli leads to death of infected rabbits in a time course similar to that seen during the bacteremic phase of intranasal- or SC-initiated infections [22]. This finding indicates that the development of bacteremia and hematogenous spread are an intermediate step in the progression of the anthrax disease, validating the significance of this assay. In addition, we observed that the results obtained with the rabbit IV inoculation assay parallel those obtained for GP infected SC. Therefore, the IV inoculation assay was used for initial characterization of the factors involved in the regulation of the toxin-independent virulence mechanism.

In both models, our results demonstrate that curing pXO1 from *B. anthracis* results in complete loss of virulence. On the other hand, deletion of the toxin genes, *pag, lef and cya*, from pXO1 of Vollum-wt (creating VollumΔ*pag*Δ*cya*Δ*lef*) results only in moderate attenuation while maintaining significant virulence. In addition, this virulence was shown to depend on AtxA activity in the presence of pXO2, as deletion of either *atxA* (VollumΔ*pag*Δ*cya*Δ*lef*Δ*atxA*) or pXO2 (VollumΔpXO2) results in complete loss of virulence.

AtxA, a global *B. anthracis* virulence regulator is a 476 amino-acid-long protein encoded by the activator gene *atxA*, located in the pXO1 pathogenicity island. Although the mechanisms by which AtxA exerts its regulation are not yet fully understood, AtxA was shown to control the expression of more than a hundred genes residing on all genetic elements (the chromosome and the two virulence plasmids) [3]. For AtxA to activate additional genes of unknown genomic location required for exerting this novel virulent trait, the prolonged survival of the bacteria in the circulation of a naïve host may be needed. This type of protection can be exerted by pXO2, through the capsule, or pXO1, through the toxins. This assumption is supported by the results with VollumΔpXO2 (**Figure 1A**), which also induces partial lethality in IV inoculated rabbits, though with longer MTTD. This demonstrates that a toxinogenic strain lacking pXO2 is sufficiently protected in the circulation by the toxins, thus remaining able to exhibit the virulent trait. On the other hand, systemic toxemia has been shown to cause death of mice, rats (Fisher) and GP [23], and therefore we cannot conclude which virulence mechanism was crucial in this strain's ability to kill the host. As the virulence exerted by the VollumΔ*pag*Δ*cya*Δ*lef* encapsulated vegetative bacilli depends both on AtxA and pXO2, we studied the effect of AtxA activity on the generation and function of the main product of pXO2, the capsule. Using a simple India-ink negative stain, no qualitative difference could be detected between the capsules of the assumed AtxA-activated bacilli and the bacteria lacking AtxA (**Figure 4**). Several studies have shown that *atxA* mutant bacilli are less or non-encapsulated *in vitro* [2], [24], however this mutation did not affect capsule production *in vivo* [25]. It seems that our *ex vivo* conditions mimic the *in vivo* situation. Variations in capsule function may result in differences in bacterial survival in the circulation. Indeed, bacteremia levels determined for encapsulated AtxA-activated strains (virulent strains) were higher than those determined for the *atxA* mutant strains (non-virulent strains, **Figure 5**). The variation in bacteremia levels in the circulation can result in differences in bacterial dissemination to the tissues. Comparison of the tissue bacterial burden shows that virulent strains seem to exhibit higher levels than the non-virulent strains (**Figure 6**). Although not statistically significant, these differences correlate with the bacteremia pattern and may indicate that the virulence of *B. anthracis* strains may be related to their ability to spread to different tissues. However, it should be emphasized that while this difference may reflect a leading cause for the development of the disease towards host mortality, it may also prove to be irrelevant. Passive immunization with sera from convalescent rabbits, while saving naïve rabbits inoculated IV with VollumΔ*pag*Δ*cya*Δ*lef* encapsulated vegetative cells, did not affect bacterial systemic spread. This indicates that tissue dissemination *per se* is not the main determinant of virulence.

The convalescent serum was further used to try and identify the antigen responsible for the protective activity. Using Western-blot, the protective sera recognized a distinct band of about 70 Kd present in the bacterial extract of the virulent strains, but not in the extract of the non-virulent strains, which was identified as pXO1-90 (BslA – *B. anthracis* S-layer protein A). BslA, a putative surface layer immunoreactive protein [26] was studied and shown to mediate adherence of the Sterne vegetative bacteria to host cells [27], [28], [29], including to the blood-brain barrier endothelial cells, promoting penetration during the pathogenesis of anthrax meningitis [30]. However, deletion of the *bslA* gene in the VollumΔ*pag*Δ*cya*Δ*lef* background had a minor effect on the pathogenicity of the mutant (**Table S1**), and no effect on tissue dissemination.

In order to evaluate the contribution of pXO1 to the toxin-independent virulence mechanism, we inserted the *atxA* gene into the genome of the VollumΔ*pXO*1 mutant, creating VollumΔ*pXO*1*BA2805::atxA*. The virulence exhibited by the new mutant indicates that AtxA, in the background of pXO2, is sufficient to induce the virulent trait. Furthermore, as deletion of *atxA* from the virulent strain VollumΔ*pag*Δ*cya*Δ*lef* results in loss of virulence, and insertion of *atxA* into the non-virulent VollumΔ*pXO*1 results in recovery of the virulent trait (**Fig 7**), we can conclude that *atxA* is the only gene from pXO1 required for the exhibition of the IV virulence.

Further studies to genetically define the toxin independent virulence should include, among other, identification of components on pXO2 that might be regulated, directly or indirectly by AtxA, such as the *capBCADE* structural genes and *acpA/acpB* regulatory genes, as well as testing additional *B. anthracis* strains and relevant animal models.

To conclude, in this study we demonstrate that the toxin-independent virulence mechanism, demonstrated previously in GP, is a general trait of *B. anthracis*. Strains lacking the three toxin-genes, previously shown to kill GP when injected SC as spores [6], were now shown to be able to kill rabbit hosts when injected IV as encapsulated vegetative cells. This mechanism of virulence was shown to be AtxA dependent. This novel virulence mechanism should be further explored, as it may prove to be a fundamental virulent trait of *B. anthracis*. The fact that artificially-induced VollumΔ*pag*Δ*cya*Δ*lef* bacteremia in animals could be cured by passive immunization indicates that bacterial antigens other than PA induce this immune response. Therefore we assume that the main findings described in this work may have major implications on future research both on *B. anthracis* pathogenicity and on vaccine development.

## Supporting Information

Table S1 **Susceptibility of rabbits to Vollum strains in the IV injection model.** Rabbits were inoculated IV with different doses of vegetative cells of the wild type and mutants strains.
(DOCX)

Table S2 **Effect of** ***atxA*** **gene on the toxin independent virulent trait in rabbits IV injection model.** Rabbits were inoculated IV with different doses of vegetative cells of the wild type and mutants strains.
(DOCX)
