# Supplementary material for: Toxin-Independent Virulence of Bacillus anthracis in Rabbits
Source: PLoS One. 2014 Jan 8;9(1):e84947. doi: 10.1371/journal.pone.0084947 (PMC3885664; doi:10.1371/journal.pone.0084947)
Supplement: Table S1 — Susceptibility of rabbits to Vollum strains in the IV injection model. Rabbits were inoculated IV with different doses of vegetative cells of the wild type and mutants strains. (DOCX) [file pone.0084947.s001.docx]

**Table S1: Susceptibility of rabbits to Vollum strains in the IV injection model**

| **Strain** | **Description** | **Inoculum**  **(CFU)** | **Dead/**  **infected** | **MTTD**  **(days)** |
| --- | --- | --- | --- | --- |
| Vollum | pXO1^+^pXO2^+^ | 10^7^ | 4/4 | 1 |
|  |  | 10^5^ | 4/4 | 2 |
|  |  | 10^4^ | 2/2 | 1-2 |
|  |  | 10^3^ | 2/2 | 2-3 |
|  |  | 10^2^ | 4/4 | 2-4 |
|  |  | 10^1^ | 3/4 | 3-4 |
|  |  |  |  |  |
| Vollum ΔpXO1ΔpXO2 | pXO1^-^pXO2^-^ | 10^8^ | 0/4 | >14 |
|  |  |  |  |  |
| Vollum ΔpXO2 | pXO1^+^pXO2^-^ | 10^7^ | 3/6 | 4-6 |
|  |  |  |  |  |
| Vollum ΔpXO1 | pXO1-pXO2+ | 10^8^ | 0/4 | >14 |
|  |  |  |  |  |
| Vollum Δ*pag*Δ*cya*Δ*lef* | Complete deletion of the *pag, lef* and *cya* genes | 10^7^ | 2/2 | 1 |
|  |  | 5x10^6^ | 4/4 | 1 |
|  |  | 5x10^5^ | 2/3 | 2.5 |
|  |  | 5x10^4^ | 0/3 | >14 |
|  |  | 5x10^4^ | 1/3 | 3 |
|  |  |  |  |  |
| Vollum Δ*pag*Δ*cya*Δ*lef*Δ***atxA*** | Complete deletion of the *pag, lef* , *cya* and *atxA* genes | 10^8^ | 0/4 | >14 |
|  |  | 10^7^ | 0/5 |  |
|  |  |  |  |  |
| Vollum **ΔpXO2**Δ*pag*Δ*cya*Δ*lef* | Complete deletion of pXO2 and the *pag, lef* and *cya* genes | 10^8^ | 0/4 | >14 |
|  |  |  |  |  |
| Vollum Δ*pag*Δ*cya*Δ*lef*Δ***bslA*** | Complete deletion of the *pag, lef* , *cya* and *bslA* genes | 10^7^ | 4/4 | 2 |

Rabbits were inoculated IV with different doses of vegetative cells of the wild type and mutants strains.
