# Supplementary material for: Toxin-Independent Virulence of Bacillus anthracis in Rabbits
Source: PLoS One. 2014 Jan 8;9(1):e84947. doi: 10.1371/journal.pone.0084947 (PMC3885664; doi:10.1371/journal.pone.0084947)
Supplement: Table S2 — Effect of atxA gene on the toxin independent virulent trait in rabbits IV injection model. Rabbits were inoculated IV with different doses of vegetative cells of the wild type and mutants strains. (DOCX) [file pone.0084947.s002.docx]

**Table S2: Effect of *atxA* gene on the toxin independent virulent trait in rabbits IV injection model**

| **Strain** | **Description** | **Inoculum**  **(CFU)** | **Dead/**  **infected** | **MTTD**  **(days)** |
| --- | --- | --- | --- | --- |
| Vollum ΔpXO1 | pXO1-pXO2+ | 10^8^ | 0/4 | >14 |
|  |  |  |  |  |
| Vollum Δ*pag*Δ*cya*Δ*lef* | Complete deletion of the *pag, lef* and *cya* genes | 10^7^ | 4/4 | 1 |
|  |  |  |  |  |
| Vollum Δ*pag*Δ*cya*Δ*lef***Δ*atxA*** | Complete deletion of the pag, lef , cya and *atxA* genes | 10^8^ | 0/4 | >14 |
|  |  |  |  |  |
| Vollum ΔpXO1 *ba2805::****atxA*** | Deletion of pXO1 and insertion of the *atxA* into the genome. | 10^7^ | 4/4 | 1 |
|  |  |  |  |  |
| Vollum **Δ*atxA*** | Complete deletion of the *atxA* genes | 10^7^ | 3/6 | 3 |
|  |  |  |  |  |
| Vollum **Δ*atxA*** SC |  | 5x10^7^ | 0/4 | >14 |

Rabbits were inoculated IV with different doses of vegetative cells of the wild type and mutants strains.
